# Application progress of ultrasound in the production and processing of traditional Chinese herbal medicines

**DOI:** 10.1016/j.ultsonch.2024.107158

**Published:** 2024-11-14

**Authors:** Ke Yang, Tao-hong Han, Yi-jun Liu, Jia-ning Zhang, Ping Zhou, Xiao-ping Yu

**Affiliations:** a Zhejiang Provincial Key Laboratory of Biometrology and Inspection and Quarantine, College of Life Science, China Jiliang University, Hangzhou 310018, China; b Center for Reproductive Medicine, Department of Obstetrics and Gynecology, Peking University Third Hospital, No. 49, Huayuan North Road, Haidian District, Beijing 100191, China

**Keywords:** Ultrasound, Chinese herbal medicines, Extraction, Drying, Cleaning, Sterilization

## Abstract

The quality of Chinese herbal medicines is the key to the quality of traditional Chinese medicine. The processing of Chinese herbal medicines is an important part of the production and quality formation of medicinal materials. Traditional processing methods have low productivity and cannot guarantee the quality of Chinese herbal medicines. Among various non-thermal processing methods, ultrasonic technology has been proved to be a very valuable green processing technology. This paper will discuss the application of ultrasonic technology in the production and processing of Chinese herbal medicines in recent years, including the extraction, cleaning, drying and sterilization of effective components of Chinese herbal medicines. This review summarizes its principle, characteristics and application progress in recent years, and discusses its existing problems. The effects of ultrasound on the chemical structure and biological activity of bioactive compounds extracted from Chinese herbal medicines are mainly introduced. In addition, this paper discusses the effects of different ultrasonic conditions such as frequency, power, time and temperature on the chemical properties and processing of Chinese herbal medicines. In general, the use of ultrasound in the production and processing of Chinese herbal medicines has great application potential.

## 1 Introduction

Ultrasound refers to sound waves with a frequency exceeding 20 kHz [1]. When ultrasonic waves propagate through a medium, their interaction with two-phase or multi-phase mediums results in the conversion of acoustic energy from the transducer into mechanical energy. This process induces vibration in the medium, leading to physical and chemical changes. Subsequently, a range of effects encompassing mechanics, thermodynamics, optics, electricity, and chemistry occur. These effects primarily manifest as cavitation, as well as mechanical and thermal influences [2], [3]. The advancement of science and technology has facilitated the integration of various technological domains, thereby enabling the widespread application of ultrasonic technology across diverse sectors such as industry [4], chemical industry [5], [6], medicine [7], [8], food processing [9] and wastewater treatment [10], [11].

The application of ultrasonic technology is generally divided into two types: high-frequency low-field strength (frequency 100 kHz to 1 MHz, field strength less than 1 W/cm^2^) and low-frequency high-field strength (frequency 20 to 100 kHz, field strength 10 to 1 000 W/cm^2^) [12]. Ultrasonic technology with high frequency and low field strength is often used in medical imaging and non-destructive testing. This technology relies on three key parameters of ultrasound (sound velocity, attenuation coefficient and acoustic impedance) to analyse the properties and structural variations of objects [13]. Low-frequency, high-field strength ultrasonic technology (10 ∼ 1000 W/cm^2^) is commonly employed for tasks such as cleaning, sterilization, and extraction of active ingredients. This technology primarily harnesses the effects of ultrasonic cavitation, mechanical impact, and free radicals. In recent years, the utilization of low-frequency and high-field-strength ultrasonic technology in traditional Chinese medicine production, processing and extraction has garnered significant interest.The utilization of ultrasonic technology in the processing of traditional Chinese medicine primarily encompasses the extraction of active ingredients, cleaning, drying, and sterilization. Ultrasonic-assisted extraction is extensively applied in extracting traditional Chinese medicine components and in sample preparation for quality assessment due to its time efficiency, high extraction rates, and energy conservation benefits [14].

In recent years, with the development of the application field of low frequency and high field strength ultrasonic technology, the application of ultrasonic wave in the production, processing and extraction of Chinese medicinal materials has attracted people's attention. The water content of Chinese medicinal materials is high after harvest, and it is easy to mildew and deteriorate without timely processing, which seriously affects the quality and clinical efficacy of medicinal materials [15], [16]. Traditional Chinese medicine processing technology generally has problems such as low process level, low efficiency, low utilization rate of medicinal materials, high energy consumption, and heavy pollution [17], [18]. This has seriously restricted the sustainable development of the Chinese medicine industry and the process of modernization and internationalization. Compared with traditional technology, ultrasonic is a green processing technology with the advantages of low energy consumption and high efficiency. This technology is widely used because it reduces the damage caused by traditional processing techniques to Chinese medicinal materials.

This paper reviews the principle and application progress of ultrasonic technology in the processing of Chinese medicinal materials (extraction, cleaning, drying and sterilization). In addition, this paper introduces the effects of ultrasound on the bioactive components and pharmacological activities of Chinese herbal medicines. It is expected to provide reference for the future research of ultrasonic technology in the field of traditional Chinese medicine.

## 2 Basic overview of ultrasound research

### 2.1 The trends of publication outputs

**Fig. 1:**
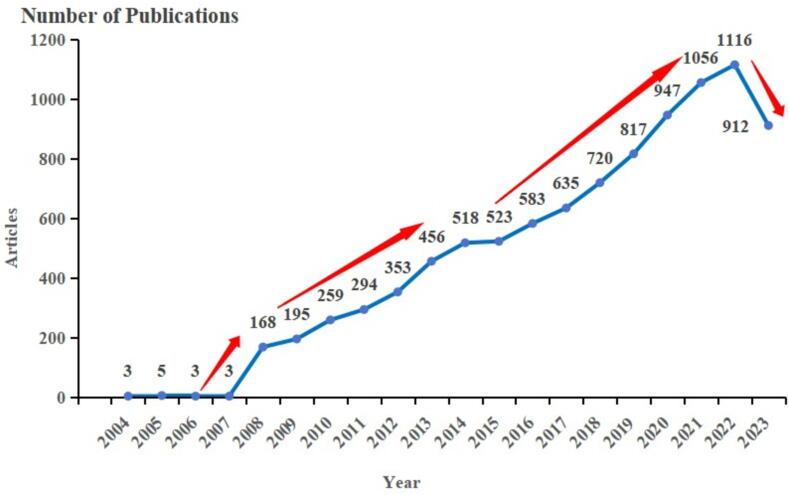
The overall trend chart of the publication.

The analysis of the number of publications in the literature is represented in the form of a line graph, which can visualize the speed and development process of ultrasonic technology research. This paper used the Web of Science database to collect data on ultrasonic technology research topics. The subject term was set to 'ultrasound' for search, and the publication date was selected as 2004–2023. A total of 9566 publications related to the field of ultrasonic technology were selected. Then, using the analysis function of WOS combined with Excel, a line chart of the number of ultrasonic technology publications was created (Fig. 1).

From 2004 to 2023, a total of 9,566 ultrasound-related articles were published, and the number of literature showed a trend of steady growth from year to year. The development of ultrasound was divided into five stages according to Fig. 1: in the initial stage of development (2004–2007), the number of annual publications was overall low, accounting for only 0.15 percent of the total. The growth stage of development (2008–2013), with an average of 288 articles per year and an average annual growth rate of 22.10 %. During this period, ultrasound research flourished, with a jump in the number of articles in 2008. The stabilization phase of development (2014–2015) averaged 521 articles per year, with an average annual growth rate of 0.97 %, and the trend of growth in the number of articles was flat. The rapid phase of development (2016–2021) accumulated 4,758 publications, an average of 793 per year, with an average annual growth rate of 12.62 %, a significant growth trend relative to the previous phase. The slight fallback period of development (2022–2023) accumulated 2,028 publications, an average of 1,014 per year. 2023 saw a decrease in the number of publications compared to the previous year, but was still in a booming phase of development. At this point, the groundwork for ultrasound had been largely completed, and it is expected that future growth in the number of publications will continue to show a steady trend. During the period of 2004–2023, the average annual number of ultrasound-related articles published is about 478, with an average annual growth rate of 35.1 %, showing a general trend of rapid development. It seemed that the research on ultrasonic waves at home and abroad maintains a high degree of enthusiasm in general.

### 2.2 Hot spot analysis of ultrasound

**Fig. 2:**
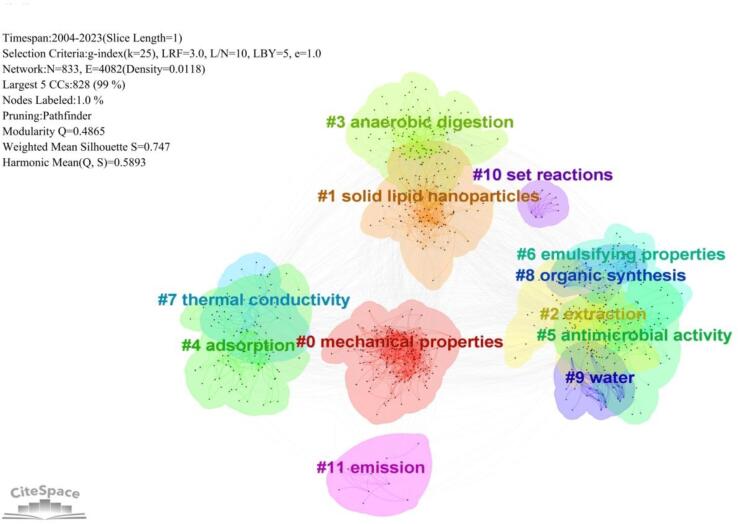
Clustering of keywords in ultrasound literature, 2004–2023.

Research hotspots are the focus of researchers in a certain field. Keyword clustering can show the development trend and research hotspots of ultrasound. This article used CiteSpace software to analyze keywords in the field of ultrasound research. Based on the Web of Science core database, the keywords of ultrasonic literature were clustered. CiteSpace uses the structure of the graph network with cluster analysis to calculate the Modularity Q value and the average profile Silhouette S value as two indicators to judge the reasonableness of the analysis. Modularity Q value between 0.3 and 0.8 is a compliant plot; when Silhouette S value is greater than 0.5 it indicates that clustering is reasonable[19]. The data in Fig. 2 showed that the Q-value of this clustering is 0.4865, which indicates that the clustering is reasonable, and the S-value is 0.5893, which indicates that the clustering results are reasonable.

The keyword clustering diagram showed 12 clustering labels (Fig. 2) respectively: #0 mechanical propertie, #1 solid lipid nanopartic, #2 extraction, #3 anaerobic digestion, #4 adsorption, #5 antimicrobial activity, #6 emulsifying propertie, #7 thermal conductivity, #8 organic synthesis, #9 water, #10 set reactions, #11 emission. With reference to the method of analyzing the clustering diagram, the analysis of the clustering labels showed that the research on ultrasound in the WOS database is broadly divided into two directions: ① physicochemical effects produced by ultrasound waves(0 mechanical propertie, #4 adsorption, 6 emulsifying propertie, #7 thermal conductivity). ② Applications of ultrasound in chemical industry, material science, medicine, sewage treatment, etc. (#1 solid lipid nanopartic, #2 extraction, #3 anaerobic digestion, #5 antimicrobial activity, #8 organic synthesis, #10 set reactions).

## 3 Application of ultrasound in the production and processing of traditional Chinese medicine

### 3.1 Extraction of active ingredients of traditional Chinese medicine

**Fig. 3:**
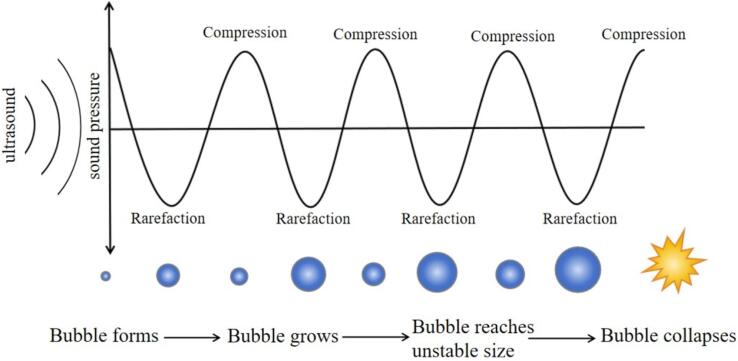
Ultrasonic cavitation phenomenon[26].

To realize the modernization and development of traditional Chinese medicine, it is necessary to improve the quality of research and development of traditional Chinese medicine, especially the use of extraction technology of active ingredients of traditional Chinese medicine is a very critical link. At present, the extraction technology of active components of Chinese medicinal materials mainly includes hot water extraction (HWE), ultrasonic assisted extraction (UAE) and enzyme-assisted extraction. In addition, there are supercritical fluid extraction and microwave-assisted extraction (MAE), etc. (Table 1). The most important mechanism of ultrasonic extraction is the cavitation effect produced by ultrasonic waves. Ultrasonic cavitation is the process by which tiny bubbles present in a liquid vibrate, grow and collapse under the action of an ultrasonic field (Fig. 3). Ultrasonic cavitation is the process by which tiny bubbles present in a liquid vibrate, grow and collapse under the action of an ultrasonic field. The extraction medium is torn into many small holes by the action of high energy ultrasonic wave. Subsequently, the small cavity is closed instantaneously, and the pressure in the high temperature and high pressure area can reach thousands of atmospheric pressure. At the same time, it is accompanied by a strong shock wave and a jet with a speed of 400 Km per hour, resulting in the destruction of plant medicinal cells. Stirring and heating during extraction can change the physical properties of cells. This increases the frequency and speed of the movement of the material molecules, and the destroyed lysozyme gradually rises. In general, ultrasound promoted the mixing of plant active ingredients and solvents, which was conducive to the extraction of active ingredients [20].

**Table 1:** Common extraction methods.

Extraction methods	Extracted material	Extraction principle	Optimal extraction conditions	Extraction rate（%）
Hot-water extraction	*Astragalus cicer L.* polysaccharides [21]	The use of hot water to disrupt plant cells, which are destroyed as the cells absorb water and swell, thus allowing the polysaccharide components to leach out.	Extraction time 61 min, water/feed ratio 25:1 mL/g, extraction temperature 75℃.	10.97
Enzyme-assisted extraction	*Equisetum arvense*total flavonoids [22]	Utilizing the specificity of enzymes, plant cell walls are broken down and destroyed by enzymes, resulting in rapid dissolution of the active ingredients in plant cells.	Cellulase concentration 0.52 %, extraction time 50.58 min, extraction temperature 49.03℃.	0.488
Ultrasonic extraction	*Auricularia auricula* polysaccharides[23]	The use of ultrasound mechanical effect, cavitation effect and thermal effect can destroy the plant cell wall, accelerate the effective ingredients in the plant cell dissolution speed.	Ultrasonic time 28.06 min, ultrasonic power 396.83 W, water/feed ratio 43.31 mL/g.	21.89
Supercritical fluid extraction	*Usnea subfloridana* usnic acid[24]	Utilizing the physical properties of supercritical fluids and the principle of extraction, the density of supercritical fluids can be drastically changed when the temperature or pressure is changed, so as to selectively extract different components in plants.	Extraction temperature 85℃, extraction time 80 min, extraction pressure 150 atm.	1.11
Microwave-assisted extraction	*Salvia miltiorrhiza* polysaccharides[25]	When microwave is added during water extraction, the polar substance will cause strong oscillation under the action of alternating electromagnetic field, which is beneficial to the rapid leaching and diffusion of polysaccharides.	Microwave power 1200, extraction time 12 min, material-liquid ratio 43.31 mL/g, ethanol concentration of 86 %.	14.11

UAE is an advanced extraction technology. Compared with the traditional extraction methods, it has the advantages of short extraction time, low temperature, high efficiency and solvent saving. Therefore, it is widely used in the extraction of effective components of traditional Chinese medicine. This study mainly analyzed the application of ultrasonic extraction method in the optimization of extraction process of polysaccharides, polyphenols and flavonoids.

#### 3.1.1 Ultrasonic extraction of polysaccharides

Polysaccharides are widely found in plants, fungi, microorganisms, algae and animals. It is a natural polymer formed by ten or more monosaccharide units linked by glycosidic bonds. Polysaccharides have antioxidant, antibacterial and antiviral properties, delayed aging, immunomodulation, tumor inhibition, hypoglycemia, hypolipidemia, etc. [27]. With the in-depth study of polysaccharides in Chinese herbal medicines, polysaccharide extraction technology has been developed continuously. Studies have shown that the extraction method will directly affect the extraction rate, monosaccharide composition, molecular weight and spatial configuration of polysaccharides. In addition, the extraction method also determines the biological activity of polysaccharides [28].

Table 2 lists the optimal process conditions for the ultrasonic extraction of polysaccharides from a variety of Chinese herbal medicines. Ultrasonic time (t), material-liquid ratio (m/v), ultrasonic temperature (T) and ultrasonic power (P) are the main factors affecting the polysaccharide extraction rate. Compared with HWE (1.28 ± 0.29 %), UAE had higher *Acanthus ilicifolius* polysaccharide yield (2.46 ± 0.43 %) and extraction efficiency [29]. The polysaccharides extracted by the two methods had the same composition of d-glucose, d-arabinose and d-galactose, but their proportions were different. UAE polysaccharides had higher d-glucose ratio and lower d-arabinose. This is because ultrasonic treatment causes the side chain of d-arabinose to break. The DPPH radical scavenging activity and scavenging hydroxyl ion activities of UAE polysaccharides were also better compared with HWE.

**Table 2:** Comparative analysis of ultrasonic extraction of plant polysaccharides.

Extracted substances (polysaccharides)	Methods of analysis	Optimal conditions for ultrasonic extraction	Extraction rate(%)	Reference
*Ligusticum* Chuanxiong Hort	Single factor experiment, orthogonal experiment	T = 80℃, t = 40 min, m/v = 1:30 g/mL	20.75	[36]
*Eucommia ulmoides*	Orthogonal experiment	P = 200 W, T = 60℃, t = 80 min	16.495	[37]
Coix seeds	Response surface method	T = 16 min, P = 480 W, m/v = 1:21 g/mL	8.340	[38]
*Angelica sinensis*	Single factor experiment, orthogonal experiment	t = 28.06 min, P = 396.83 W, m/v = 1:43.31 g/mL	21.89	[39]
*Hemerocallis citrina*	Response surface method	P = 694 W, T = 71℃, t = 38 min, m/v = 1:25 g/mL	15.25	[40]

It is reported that ultrasonic treatment will lead to a decrease in the molecular weight of polysaccharides. This is because the distribution of long-chain polymers during ultrasonic treatment moves from a larger Mw region to a smaller Mw region. For example, *Ganoderma lucidum* polysaccharides extracted utilizing UAE resulted in an MW of 465.65 kDa compared with 703.45 kDa of HWE [30]. The MW of UAE and HWE *Platycodon grandiflorum* polysaccharides were 3.14 kDa and 3.44 kDa. And Fourier transform infrared spectroscopy (FT-IR) showed that both polysaccharides are pyranose rings with α- and β-glycosidic bonds [31]. The molecular weight of *Plantago asiatica L.*seeds polysaccharide decreased from 3870 to 651 kDa after ultrasonic treatment for 2 min [32]. In addition, the apparent viscosity of polysaccharides also decreased with the prolongation of ultrasonic treatment time. However, FT-IR showed that the structure of the polysaccharide did not change significantly.This conclusion shows that ultrasonic treatment leads to the destruction of aggregation between polysaccharide molecules, rather than the destruction of polymer chains.

In addition, ultrasound-assisted enzymatic extraction is an effective method to enhance the activity of natural polysaccharides. Wei et al. [33] found that polysaccharide from the leaves of *Cercis chinensis* Bung extracted by ultrasonic had higher DPPH, hydroxyl radical scavenging ability and reducing ability. Guo et al. [34] found that ultrasonic extraction of polysaccharides had significant immune activity, and the promotion of TNF-α and IL-6 release in RAW 264.7 cells was better than that of hot water extraction of polysaccharides. Yu et al. [35] found that compared with the traditional hot water extraction method, the ultrasound-assisted extraction of *Imperata cylindrica* polysaccharide showed stronger antioxidant activity and improved the ability of cell damage caused by uric acid stimulation.

In summary, the biological activity of polysaccharides is mainly affected by many factors, such as molecular weight, uronic acid content, polysaccharide content and so on. These factors are related to the structural differences caused by extraction methods or different extraction conditions. Excessive ultrasonic power, frequency and duration may lead to the loss or degradation of bioactive components. Therefore, in the UAE process, wise parameter selection must be made according to specific conditions to ensure maximum bioavailability.

#### 3.1.2 Ultrasonic extraction of polyphenols

Plant polyphenols have phenolic functional groups and are a class of secondary metabolites with polyphenol structure widely present in plants. The unique structure of plant polyphenols makes it have many effects. For example, antibacterial, anti-allergic, anti-mutagenic, anti-cancer, anti-tumor and anti-aging and other biological activities [41]. Polyphenols are widely present in food, but they cannot be synthesized by the human body itself and can only be extracted from plants. At present, the conventional extraction techniques of polyphenols include traditional extraction methods such as decoction, reflux and soxhlet extraction. However, these methods require time-consuming and expensive equipment, as well as high purity solvents [42]. The extraction efficiency of ultrasonic method is obviously better than that of traditional solvent extraction method, and it has been greatly improved in terms of polyphenol yield and energy saving.

Other than water, ethanol and methanol solution have been utilized as the solvent for UAE based polyphenol extraction from Chinese herbal medicine, such as Sargassum muticum [43], Blackthorn Flower [44], Garcinia indica [45] and others. Studies have shown that aqueous organic solvents have higher extraction efficacy compared to low concentration and absolute organic solvents.

The application of ultrasonic extraction method to extract different polyphenols is shown in Table 3. It can be seen from the table that ultrasonic power, time and temperature are the main factors affecting the extraction rate of polyphenols. Studies have shown that the increase of ultrasonic power promotes the transfer, diffusion and dissolution of polyphenols in cells. However, excessive ultrasonic power may lead to the collapse of cavitation vesicles, resulting in higher pressure. This will accelerate the degradation of polyphenols [46]. For example, Sun et al.found that when the ultrasonic power exceeded 80 W, the extraction rate of *Areca catechu L.*seed polyphenols began to decrease [47]. It is reported that too high temperature will cause the hydroxyl groups of phenolic compounds to undergo oxidative condensation reactions. Therefore, ultrasonic extraction of polyphenols at low temperature can avoid degradation and improve the extraction rate [48]. Gueffai et al. [49] found that the extraction rate of black cumin seeds polyphenols increased gradually in the range of 40−50℃. When the optimum temperature was over 60℃, the extraction rate decreased significantly. Studies have shown that the extension of time can improve the extraction efficiency by completely breaking the plant cells and can also make the solvent diffuse to dissolve the phenolic compounds. However, polyphenols are degraded due to the effects of light, oxygen and heating due to prolonged extraction [50]. Black cumin seeds polyphenol extraction rate increased when the extraction time increased from 15 to 30 min. However, the extraction rate of polyphenols began to decrease after the extraction time exceeded 30 min [49].

**Table 3:** Comparative analysis of ultrasonic extraction of plant polyphenols.

Extracted substances (polyphenols)	Extraction solvent	Optimal conditions for ultrasonic extraction	Extraction rate(%)	Reference
*Areca catechu L.* seeds	65 % ethanol（v/v）	P = 87 W, T = 62℃, t = 153 min	13.962	[47]
*Boletus bicolor*	42 % ethanol（v/v）	t = 41 min, T = 40℃, m/v = 1:34 g/mL	1.369	[53]
*Ilex latifolia*	53 % ethanol（v/v）	T = 60 ℃, t = 26 min, m/v = 1:60 g/mL	3.577	[54]
Thinned peach	Water	t = 25 min, T = 50℃, P = 147 W, m/v = 1:12 g/mL	0.159	[55]
Green tea	ChCl-based deep eutectic solvents	P = 461.5 W; t = 21 min, m/v = 1:36 g/mL	24.3	[56]
*Picea abies*	53 % methanol （v/v）	T = 63℃, m/v = 1:38 g/mL	1.106	[57]

In summary, exploring the optimal process conditions for ultrasonic extraction can minimize the destruction of polyphenol heat-sensitive components. At the same time, less time is used to produce higher extraction rate. Nafar et al. [51] found that the importance of factors affecting bioactive compounds can be ranked in the following order: ultrasonic frequency > temperature > extraction time. On the other hand, compared with the traditional extraction method, the polyphenol content of picea abies bark extracted by ultrasound was 1.1 to 7.1 times higher. Upadhyay et al. [52] compared the effects of ultrasound-assisted extraction (30 kHz, 40℃, 15 min) and conventional solvent extraction on the extraction of polyphenols from Ligusticum chuanxiong leaves. The results showed that the yield of polyphenols extracted by ultrasound-assisted extraction (6.83 mg GAE/g) was higher than that of conventional solvent extraction (3.84 mg GAE/g). In short, ultrasonic extraction time is short, the extraction rate is high, is the preferred method of polyphenol extraction.

#### 3.1.3 Ultrasonic extraction of flavonoids

Flavonoids are hydroxylated phenolic compounds that usually exist in the glycoside or free state, especially in the leaves, roots, stems and peels of plants with high content. They have biological activities such as antioxidant, antibacterial and anti-inflammatory, blood lipid regulation and anti-cellular aging effects. At present, domestic and foreign methods for extracting flavonoids mainly include direct solvent extraction, ultrasonic extraction and supercritical CO_2_ extraction. Each of these methods has some advantages and disadvantages, but none of them has a high extraction rate. Ultrasonic waves use the cavitation effect to increase the penetration of the solvent, making the flavonoids more soluble in the extraction solvent, thus improving the extraction efficiency [58], [59]. The application of flavonoid extraction using ultrasonic extraction is shown in Table 4.

**Table 4:** Comparative analysis of ultrasonic extraction of flavonoids.

Extracted substances (flavonoids)	Extraction solvent	Optimal conditions for ultrasonic extraction	Extraction rate (%)	Reference
Abrus cantoniensis	50 % ethanol（v/v）	t = 40 min, m/v = 1:47 g/mL, P = 125 W, extraction cycles = 4	3.68	[68]
*Tussilago farfara L.*	Water	t = 30 min, P = 420 W, m/v = 1:25 g/mL	6.59	[69]
*Xanthoceras sorbifolia* bunge flowers	80 % ethanol（v/v）	T = 84℃, t = 1h, m/v = 1:37 g/mL	3.956	[70]
*Astragalus membranaceus* stems and leaves	75 % ethanol（v/v）	T = 58℃, t = 35 min, m/v = 1:40 g/mL	2.203	[71]
*Radix Puerariae*	59 % ethanol（v/v）	t = 43 min, m/v = 1:40 g/mL	2..082	[72]
*Camellia fascicularis* leaves	40 % ethanol（v/v）	T = 72.3℃, t = 1.6 h, m/v = 1:60 g/mL	4.765	[73]

In general, organic solvents, water and a mixture of these solvents are usually used to extract flavonoids from Chinese herbal medicines. UAE widely uses organic solvents such as ethanol, methanol, acetone and isopropanol to mix different proportions of water to extract flavonoids from plants [60]. In addition, UAE combined with ionic liquid is a new extraction method with high efficiency, environmental protection and low energy consumption. Compared with traditional solvents, ionic liquids can quickly penetrate into plant cells and accelerate the dissolution of flavonoids. Zuo et al. [61] used ultrasound combined with ionic liquid to extract the yield of flavonoids from Pinus massoniana was 41.07 mg/g. Compared with water solvent, the extraction rate of this method was increased by 60 %.

Compared with HWE (169.64 mg/g), the total flavonoid content of *Nymphaea lotus L.* increased by 1.35 times under the optimized UAE (235.45 mg/g) [62]. Studies have shown that the yield and antioxidant activity of flavonoids extracted from Passion Fruit Peels by UAE (25.79 mg/g) were higher than those of MAE (8.11 mg/g) [63]. In addition, experiments have shown that the best solvent for Passion Fruit Peels flavonoids is a volume ratio of ethanol, water, and acetone of 0.29: 0.34: 0.37. Under these conditions, suitable polarity conditions were provided for flavonoids. Zhao et al. [64] compared the extraction of flavonoids from *Millettia speciosa* by Soxhlet extraction, UAE and MAE. The results showed that UAE (2.083 ± 0.112 %) had the highest yield of flavonoids from *Millettia speciosa*.

In addition, with the gradual research of ultrasonic technology, there are more and more methods of extracting flavonoids by ultrasonic combined with other technologies. For example, ultrasonic-microwave synergy [65], ultrasonic-enzyme assisted synergy[66], ultrasonic-electrostatic field synergy [67]. At present, ultrasonic extraction is mainly used in laboratory. In order to be applied to large-scale industrial production, it is necessary to solve the problem of industrial equipment amplification. Ultrasonic-assisted extraction is fast, cheap and high extraction rate. It can be called a 'green technology' in line with sustainable development and environmental protection. With the further understanding of ultrasonic extraction technology, its application prospect in the extraction of flavonoids will be broader.

### 3.2 Cleaning of Chinese herbs

Cleaning is the first part of herbal medicine processing, and is also an important part that affects the quality of subsequent processing. In particular, root herbs, which are very common in cultivation, have their medicinal parts in direct contact with the soil, with relatively rough surfaces and soil, germs, etc., hidden between the branches, which must be cleaned before subsequent processing. Thus, in the cleaning and washing of Chinese herbal medicines, there is a need to explore a technology that can minimise the loss of active ingredients, remove adhering soil, and kill insect eggs and pathogenic microorganisms. On this basis, it guarantees the advantages of safety and non-pollution, reduced water consumption and lower labour intensity [74].

#### 3.2.1 The principle of ultrasonic cleaning

**Fig. 4:**
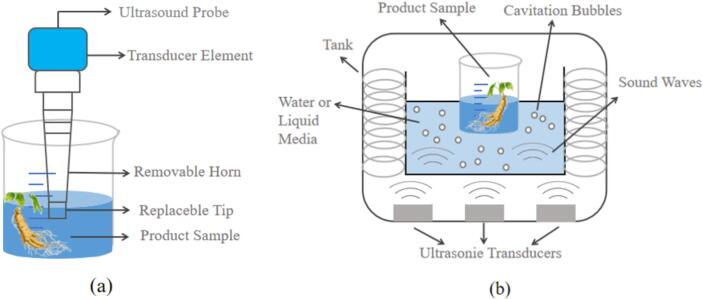
Common types of ultrasound:(a) probe type ultrasonic; (b)water bath type ultrasonic[77], [78].

Ultrasonic cleaning technology was realized in the 1950 s and is the largest branch of power ultrasound. Ultrasonic cleaning process mechanism research and equipment development in China has nearly 50 years of history, almost synchronised with foreign countries. Typically used ultrasonic instruments can be categorized into two types: probe type ultrasonic and water bath type ultrasonic (Fig. 4). The principle of ultrasonic cleaning is that the ultrasonic waves generated in the water produce enough “cavitation bubbles” and release energy to complete the cleaning operation. When ultrasound propagates in a liquid medium, it generates mechanical vibrations and acoustic currents, thus converting acoustic energy into mechanical energy [75]. Gases dissolved in a liquid medium expand under the action of ultrasound, completing a series of reactions such as the formation and rupture of cavitation bubbles. At the same time, cavitation caused by temperature and pressure changes can also cause chemical changes that accelerate cleaning efficiency [76].

#### 3.2.2 Application of ultrasonic cleaning Chinese medicinal materials

**Fig. 5:**
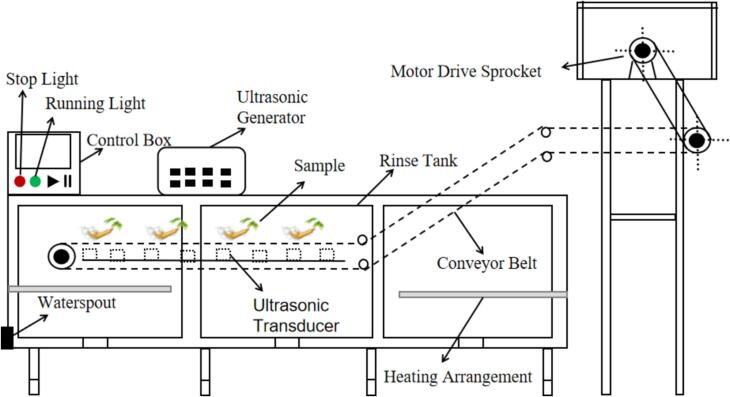
Structure diagram of ultrasonic cleaning machine.

Ultrasonic cleaning technology is widely used in various fields in recent years because of its high cleaning efficiency, fast speed and non-polluting characteristics, and it is also gradually applied in the field of Chinese herbal medicine cleaning [79]. Sun [80] and Chen et al. [81] analyzed the feasibility of applying ultrasonic technology to Chinese herbal medicine cleaning based on theory. Combined with the advantages of ultrasonic cleaning and the status quo of Chinese herbal medicine cleaning. They theoretically analyzed the practical application value of ultrasonic cleaning of Chinese herbal medicine and the focus of the proposed breakthrough. This provided a reference for the research on rapid, water-saving and pollution-free cleaning of rhizome Chinese herbal medicines. The structure of ultrasonic cleaning machine is shown in Fig. 5, which is mainly composed of ultrasonic generator, ultrasonic transducer, control box, conveyor belt, cleaning tank and heating device.

The main influencing factors of ultrasonic cleaning efficiency are ultrasonic frequency, power, cleaning time and temperature. Studies have shown that excessive ultrasonic power and long cleaning time can easily destroy the cell tissue of medicinal materials. This will cause the destruction and loss of effective components of Chinese herbal medicine. Excessive cleaning temperature may cause the loss of water-soluble active ingredients in Chinese medicinal materials. The longer the cleaning time is within a certain range, the better the cleaning effect is, but the time is too long, which may cause the loss of functional components and energy waste in Chinese herbal medicines. In order to avoid adverse effects, it is necessary to scientifically and rationally select the ultrasonic process parameters in the cleaning process. Huang et al. [82] determined the ultrasonic cleaning method of *Angelica sinensis* by orthogonal experiment. When the ultrasonic frequency was 45 kHz, the power was 300 W, and the cleaning time was 20 min, the active ingredient content of *Angelica sinensis* was the highest and significantly higher than that before cleaning. In addition, Li et al. [83] compared the effect of ultrasonic cleaning and ordinary water washing on *Angelica sinensis.* There was little difference in the content of angelica polysaccharide between ultrasonic cleaning and ordinary water washing. However, ultrasonic cleaning is more time-saving and water-saving, and has higher cleanliness. Zhang et al. [84] determined the optimal cleaning parameters of gymnadenia conopsea by using the cleanliness index and the damage rate of medicinal materials as the evaluation indexes: ultrasonic power was 600 W, frequency was 25 kHz, and cleaning time was 15 min. Under this condition, the cleanliness index of palm ginseng was 0.96. Studies have shown that the most important factor affecting the cleaning rate and damage rate of dried samples is ultrasonic power, and other factors have no significant effect on the damage rate.

Frequency is an important parameter of ultrasonic effect on cavitation process. Studies have shown that multi-frequency ultrasound can enhance the cavitation effect. Multi-frequency ultrasound can complete multi-mode ultrasonic cleaning, and set different frequencies or frequency combinations, dual-frequency or multi-frequency continuous operation, and working interval ratio. Alenyorege et al. [85] developed a new multi-frequency multi-mode ultrasound device that can work at single-frequency (20 kHz), dual-frequency (20–40 kHz), or triple-frequency (20–40−60 kHz) settings. Compared with single-frequency and dual-frequency, triple-frequency ultrasound has more advantages in the microstructure integrity and microbial inactivation of samples. The reduction of bacteria, yeast and mold was >1.5 log CFU/g. In addition, the total phenol content and DPPH free radical scavenging activity of the samples were increased by tri-frequency ultrasonic treatment. Qu et al. [86] found that compared with the traditional cleaning process, low dual-frequency ultrasound (20 + 28 kHz) combined with NaClO cleaning saves 19.99 % of water and 68 % of NaClO consumption. In addition, the retention rate of ascorbic acid, allicin and other nutritional indicators in welsh onion was higher. Compared with the scanning frequency of 28 ± 2(26–30 kHz), the TPC of the sample treated at a fixed frequency of 40 kHz increased by 13 %. The DPPH free radical scavenging activity of dried samples was affected by frequency change and combination, and the order was fixed frequency > fixed combination frequency > scanning frequency. The overall optimal reduction rates of the number of E.coli by scanning, fixed and combined fixed-frequency ultrasonic washing treatments were 2.67, 3.37, 1.80 log CFU/g, respectively. In summary, the selection of appropriate ultrasonic frequency conditions based on the characteristics of Chinese medicinal materials is essential for maintaining or improving the physical and chemical quality of dried products and consuming less energy in the manufacturing process, especially in large-scale industrial production.

In general, for different cleaning objects, the factors affecting the ultrasonic cleaning effect mainly include ultrasonic power, frequency and cleaning time. The application of ultrasonic cleaning technology in the cleaning and processing of Chinese herbal medicine has opened up an effective way for the rapid, water-saving and pollution-free cleaning of Chinese herbal medicine. This will promote the current cleaning and processing of Chinese medicinal materials from traditional empirical to scientific, from extensive to intensive, ecological green modernization direction.

### 3.3 Drying of Chinese herbs

Chinese herbal medicines need to undergo primary processing after harvesting, of which drying is a common unit operation in the processing and production of Chinese herbal medicines. The main purpose of drying is to reduce the moisture content of herbal medicines, intermediates and preparations for transportation, storage or the next manufacturing process. At present, China still mostly adopts traditional natural drying methods such as sun-drying and shade-drying to dry Chinese herbal medicines. The traditional drying method is inefficient, there is a small output, low efficiency, easy to pollute, labor intensity, can not be precisely controlled, and can not achieve standardized production [87]. In order to enhance the drying effect of Chinese herbal medicines, various modern drying technologies have been successively applied to the drying and processing of Chinese herbal medicines, which mainly include hot air drying, heat pump drying, infrared drying, ultrasonic drying and joint drying and other equipments [88].

#### 3.3.1 The principle of ultrasonic drying

**Fig. 6:**
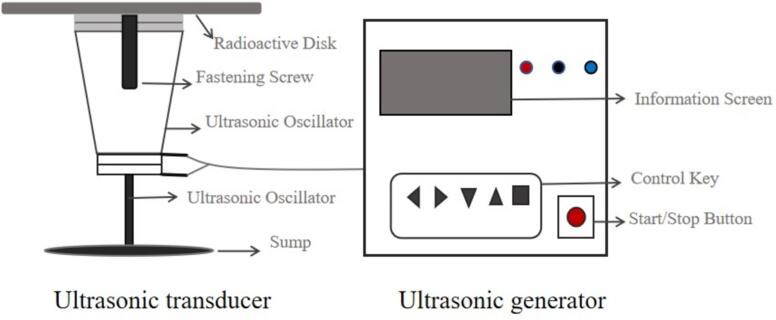
Ultrasonic generation system[91].

Ultrasonic drying technology was first reported in the 1950 s in BOUCHER's drying test using audible sound waves and ultrasonic waves [89]. Ultrasonic drying mainly uses cavitation effect, sponge effect, mechanical effect, thermal effect and other physical phenomena affecting the surface and internal structure of materials. By enlarging the cell pores, the resistance to moisture migration is reduced, which in turn improves the heat and mass transfer efficiency in the drying process and shortens the drying time. Ultrasonic drying equipment is an important carrier for the application of ultrasonic drying technology, and its core is the ultrasonic generation system [90]. From the point of view of energy conversion, the essential function of the ultrasonic generation system is to convert electrical energy into acoustic and mechanical energy, which mainly consists of ultrasonic transducers and ultrasonic generators (Fig. 6). The ultrasonic generator is driven by an alternating current that converts the output of the built-in amplifier circuit into a high-frequency alternating current signal that matches the ultrasonic transducer.

#### 3.3.2 Ultrasonic pretreatment drying technology

The drying process is closely related to the pretreatment link. Pretreatment can change the organisational structure of the material, improve the permeability of the cell membrane, and promote the diffusion of water and material flow within the material. Selection of appropriate pretreatment before drying helps to improve the drying rate and reduce the adverse effects of the drying process on the quality of the material. At present, the commonly used drying pretreatment methods include: ultrasonic pretreatment (UP), infiltration pretreatment, and needling pretreatment [92]. UP refers to the treatment method of soaking Chinese medicinal materials and other materials in solvent for ultrasonic bath before drying. Usually, the ultrasonic equipment is directly used to pretreat the material, and then the appropriate drying equipment is selected for drying.

Table 5 lists the application of UP combined with other drying equipment in the drying of Chinese herbal medicines. It can be seen in the table that the cavitation generated by the ultrasonic wave produces a strong shear force and changes the tissue structure of the plant. Microchannels and porous structures were formed in the samples after UP, which accelerated the water diffusion rate and shortened the drying time. In addition, UP can enhance the high-frequency vibration and turbulence inside the sample, which can effectively reduce the mass transfer resistance inside the material. This is conducive to the outward migration and diffusion of internal water.

**Table 5:** Application of UP in the drying of Chinese herbal medicines.

Drying technology	specimen	UP parameter settings				Main conclusions
		Frequency (kHz)	Power (W)	Time (min)	Temp (℃)	
Far-infrared drying	Licorice[100]	40	60	40	—	The moisture content of the licorice was significantly reduced.UP improved the quality of the licorice tablets and significantly reduced the time required for subsequent drying.
Electrohydr-odynamic drying	Goji berry[101]	—	200	20	35	Significantly increased drying rate, effective water diffusion coefficient and rehydration rate. The cavitation bubbles generated by UP caused the surface of wolfberry to be extremely irregular. The thickness of the surface layer of the wolfberry is reduced, which is beneficial to improve the drying speed.
Radio frequency vacuum drying	*Codonopsis pilosula* Slices[102]	20	60	30	—	Effectively improve the drying rate. The retention of polysaccharides, total phenols, flavonoids, and syringin was increased. Antioxidant capacity was significantly higher than that without UP. The cavitation phenomenon leads to the change of microstructure and the decrease of water diffusion boundary layer.
Far-infrared vacuum drying	Cistanche slices[103]	40	210	35	—	The overall structure of the sample after UP is honeycomb, which improves the drying rate and rehydration ability, and improves the drying quality of the product. The internal structure of the product dried over 45 min was seriously damaged, the surface collapsed and deformed seriously, and the content of active ingredients was low.
Hot-air drying	*Calophyllum inophyllum L.*[104]	—	288	30	—	UP produced microscopic channels and folds inside the crabapple fruit slices. It changed the internal moisture state and distribution, and accelerated the migration and removal of moisture in the hot air drying stage. The drying rate increased by 19.17 % ∼ 391.23 %.

Single hot air drying has the problems of low drying efficiency, long drying time, high energy consumption and poor drying quality. The addition of UP before hot air drying reduced the hot air drying temperature, which greatly reduced the loss of effective components of Chinese herbal medicines. For example, *Hibiscus sabdariffa L.* [93], *Boletus aereus* [94]*,* Tartary buckwheat sprouts [95]. Huang et al. [96] studied the effect of ultrasonic pretreatment on hot air drying of *Phyllanthus emblica*. Compared with the control group, the energy consumption was reduced by 17.9 %. In addition, the total color of *Phyllanthus emblica* changed little and the rehydration rate was high after ultrasonic pretreatment. This is because the structural damage during the ultrasonic pretreatment increases the water absorption during the rehydration process. Under the action of ultrasonic wave, the micro-disturbance formed near the surface of *Phyllanthus emblica* reduced the thickness of the diffusion boundary layer. Rapid compression and expansion make the microchannel suitable for fluid movement, and the internal resistance of mass transfer decreases, thus increasing water migration.

Studies have shown that microwave drying alone can cause uneven drying and surface carbonization of Chinese medicinal materials due to excessive heating, which seriously affects the quality of dried samples. The cavitation generated by UP promotes the formation of microchannels. This is beneficial to accelerate the heat and mass transfer during the microwave drying process of the sample, promote water migration, and improve the dehydration rate of the dried material. In short, UP can effectively solve the problem of uneven microwave drying. Sledz et al. [97] found that the time of microwave drying basil leaves after 35 kHz, 20 min UP was shortened by 20 %, and the total energy consumption was reduced by 26.2 %. UP changed the microstructure of basil leaves, which showed that the cell space became larger and longer. In addition, compared with the untreated samples, the phenolic content in the leaves after UP was significantly increased.

Far-infrared drying has the advantages of low energy consumption, uniform temperature distribution and good product quality. Therefore, it is widely used in the drying of Chinese herbal medicines. However, it has a weak effect on the internal mass transfer of the material. UP can improve the infrared drying process, and the two can complement each other. Pei et al.[98] found that the infrared drying time of *Crocus sativus L.*was shortened by 21.05 % after 60 s UP at 50℃.UP can significantly shorten the drying time and improve the color quality. This may be due to the generation of microchannels on the surface of saffron, which reduces the resistance to water migration. Zhang et al. [99] found that UP can promote the far-infrared drying of ginger slices. This is because ultrasonic pretreatment can change the microstructure of ginger slices. The cavitation and mechanical action of ultrasonic wave produced many micro-channels inside the ginger slices, which made the moisture of ginger slices easily and quickly removed during the drying process. Therefore, the far-infrared drying rate of ginger slices was accelerated and the drying time was shortened. In general, the rapid shrinkage and expansion of ultrasonic waves inside the dried sample is conducive to the internal water diffusion of Chinese medicinal materials. This can effectively improve the dehydration rate of infrared drying and improve the quality of dried products.

In summary, the quality of dried products using ultrasonic as a pretreatment technology is better than that of Chinese medicinal materials without pretreatment. The unique mechanical effect and cavitation effect of ultrasound reduce the mass transfer resistance inside the material, improve the drying rate of Chinese medicinal materials, and shorten the drying time. Ultrasound as a pretreatment before drying of Chinese herbal medicines can effectively reduce the drying process and improve product quality. Although a lot of research results have been achieved in the field of ultrasonic pretreatment drying, more research and technology are needed to expand industrial applications. In the future application of ultrasonic technology in the drying of Chinese medicinal materials, the optimization of ultrasonic pretreatment parameters should be considered first to reduce the damage of ultrasonic pretreatment to the effective components of Chinese medicinal materials.

#### 3.3.3 Ultrasonic drying equipment

**Fig. 7:**
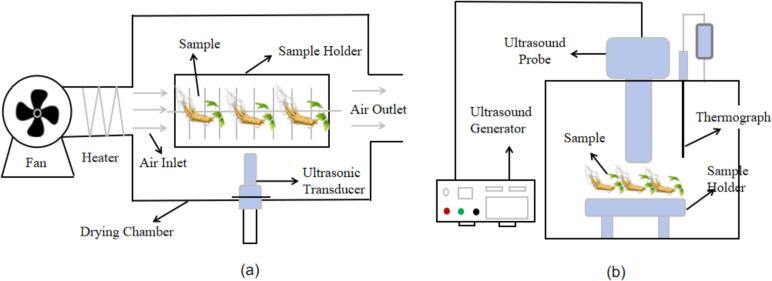
The type of ultrasonic drying equipment: (a) air-mediated ultrasonic drying; (b) Direct contact ultrasonic drying.

There are two different modes of ultrasonic energy transfer between the ultrasonic transducer and the material to be dried. They are airborne ultrasonic drying Equipment (AUD) and direct-contact ultrasonic drying technology (DUD)(Fig. 7)[105]. AUD is ultrasonic waves generated by the ultrasonic transducer, through the air propagation to the material to be dried to promote drying, including hot air equipment and ultrasonic equipment. DUD refers to the transducer and the material in direct contact or the ultrasonic energy coupled into the sample through a solid medium.

It was reported that AUD can shorten the drying time and improve product quality at low air flow rate and low temperature. DUD can only significantly improve the quality of dried products. Studies have shown that AUD has a microfluidic phenomenon during the drying process. And this phenomenon is conducive to maintaining the microstructure of the material. Compared with the absence of ultrasound, the cell deformation and structural collapse of apple slices treated with AUD were less. In addition, the moisture content of apple slices is significantly reduced, and the drying rate can be rapidly improved in a short time [91]. The drying properties of AUD and DUD were compared by drying blackberries. The results showed that both accelerated the drying process and reduced energy consumption compared with air drying alone. The retention of anthocyanins and organic acids in blackberry was enhanced. It is worth noting that the total energy consumption of contact ultrasonic assisted air drying is 27.0 % lower than that of air-mediated ultrasonic drying. After drying, the material contains more anthocyanins and organic acids [106].

In recent years, ultrasonic direct contact has played an important role in improving the drying process due to its small energy attenuation. It can reduce the cell surface damage of dried samples and the mass transfer resistance during drying. It can also increase the retention rate of bioactive substances in the sample, thereby improving the antioxidant activity of the sample. Drying by direct contact ultrasonic technology can retain more nutrients and bioactive substances, so that the dried samples have better performance in quality and function. In addition, compared with direct contact ultrasonic drying equipment, air-mediated ultrasonic drying equipment does not require direct contact with the material to be dried. The ultrasonic generator can be more easily installed in the appropriate position, and is not limited by the shape and quantity of the material, which is conducive to the mass production of ultrasonic dried Chinese medicinal materials.

In general, ultrasonic drying has certain advantages in the improvement of apparent properties and internal texture of materials and the retention of active ingredients. However, the drying process of traditional Chinese medicine is a complex heat and mass transfer process. Before drying, the appropriate drying process should be selected according to the complex components of different Chinese medicinal materials. Avoid the phenomenon of degradation and transformation of complex components of traditional Chinese medicine under the action of ultrasonic energy. At present, there are still manufacturing problems such as technology and equipment innovation in ultrasonic drying. Ultrasonic transducers for different Chinese herbal medicines were developed by studying the propagation characteristics of ultrasonic waves. This is the key to the application of ultrasound in the field of Chinese herbal medicine drying. This will be conducive to the improvement of quality and efficiency and large-scale application of ultrasonic drying technology in the drying process of Chinese medicinal materials.

#### 3.3.4 Coupling of ultrasound with other drying equipment

In order to expand the scope of application of ultrasound in the field of drying and improve its drying effect, the ultrasonic drying directly coupled with modern drying technology is currently more widely used in industrial production drying. Adopting the processing method combined with other drying technologies can further improve the quality of dried products and shorten the drying time [107]. Such as ultrasound-hot air drying, ultrasound-far infrared drying, ultrasound-vacuum drying, ultrasound-freeze drying and ultrasound-heat pump drying. Combined drying can make up for the defects of single drying and achieve the drying purpose of high drying efficiency and good quality.

The chemical properties of Chinese medicinal materials in ultrasonic coupling drying include active compound content and antioxidant activity. According to the analysis of Table 6, the influence of ultrasonic and other equipment coupling drying on the effective components of Chinese medicinal materials is analyzed. Bioactive components are important indicators for evaluating the drying quality of Chinese medicinal materials. Such as polysaccharides, total phenols and total flavonoids. Zang et al. [108] found that compared with the samples without ultrasonic treatment, the drying time of Angelica sinensis was shortened by 18.2 % − 50.0 % after ultrasonic-assisted far-infrared drying. The total phenol, total flavonoids, polysaccharide content and antioxidant capacity of Angelica sinensis were significantly improved. Compared with no ultrasound, the polyphenol content increased by 2.03 %, 12.54 %, 26.06 % and 22.91 % when the ultrasonic power was 36 W, 48 W, 60 W and 72 W, respectively. The content of polysaccharides was increased by 27.83 % compared with that without ultrasonic treatment. Wang et al. [109] found that the drying time of ultrasonic synergy vacuum far-infrared drying (US-VFID) was 25 % shorter than that of VFID. Compared with HAD, the total phenolic content and antioxidant activity of flowers treated with US-VFID increased by 29.47 % and 8.67 %, respectively.

**Table 6:** Relevant research on ultrasonic coupling other drying equipment.

Drying technology	Chinese medicinal materials	Technological parameter	Main conclusions
Ultrasonic-assisted Vacuum Far-Infrared drying	Cistanche slices[112]	Radiation temperature 55℃, ultrasonic frequency 60 kH, ultrasonic power 96 W.	Increase drying speed and improve drying quality. The maximum contents of Catapol, Leonurusoside, Rhodioloside, Echinacoside, Poliumoside, V erbascoside and Isoacteoside increased by 47.84 %, 47.47 %, 37.06 %, 34.62 %, 24.2 %, 26.43 % and 32.49 %, respectively.
Ultrasonic-assisted heat pump drying	Bo chinh ginseng[113]	Drying temperature 45.2℃, ultrasonic power 127.7 W, intermittency ratio of ultrasound generator of 0.18.	The increase in drying air temperature and ultrasonic power significantly shortened the drying time, and the effective diffusion coefficient of moisture increased significantly. The active ingredients and antioxidant activity increased with increasing ultrasound frequency.
Ultrasonic-assisted vacuum drying (UAVD)	*Schisandra chinensis*[114]	—	The drying time with UAVD was reduced by more than 25 % compared to conventional vacuum drying(CVD). The effective moisture diffusivity (D_eff_) increased by 112.93 %. The content of Schisandrol A in the extracts obtained from UAVD was 12.79 % higher than that obtained using CVD at 90 °C.
Ultrasonic far-infrared synergistic drying	*Lycium barbarum L.*[115]	Irradiation height 250 mm, ultrasonic frequency 40 kHz, ultrasonic power 127.7 W.	The application of ultrasound improves the drying rate. Compared with natural drying, the polysaccharide content was increased by 33.2 % and the total phenolic content by 44.9 %. The antioxidant activity was the strongest, and the total flavonoids content was the highest (2.594 mg/g) at 24 W ultrasonic power.
Ultrasonic-assisted hot air drying	*Flos Lonicerae*[116]	—	The application of ultrasound has a significant impact on the hot air drying(HAD) process, which is favorable to shorten the drying time and increase the drying speed of the traditional HAD process.

The above analysis shows that ultrasonic coupling with other drying equipment can improve the chemical properties of dried Chinese medicinal materials. However, in some cases, it may have adverse effects. Feng et al. [110] found that compared with single far-infrared drying, ultrasonic and far-infrared combined drying accelerated the free water mobility of ginger (7.1 ∼ 38.1 %) and shortened the drying time (280 ∼ 160 min). However, after ultrasonic intervention, the gingerol content of ginger slices with 4 mm thickness decreased by 0.1226 mg/g ∼ 0.1177 mg/g. Vallespir et al. [111] found that the loss of active ingredients and antioxidant activity (39–54 %, 57–69 %) of samples dried at 15℃ was significantly higher than that of ultrasound-coupled low-temperature (14–43 %, 23–50 %) drying at 15℃.

In general, the combination of ultrasonic and other drying techniques at an appropriate temperature can improve the drying effect. This can reduce the negative impact of ultrasound itself on the active ingredients of Chinese herbal medicines. Ultrasonic wave has an important influence on the drying efficiency and drying quality of Chinese medicinal materials. For example, shortening drying time, reducing energy consumption, and improving the bioactive substances and antioxidant activity of Chinese herbal medicines. This is beneficial to the drying of Chinese herbal medicines.

### 3.4 Anti-mold sterilization of Chinese herbal medicines

The main reason for the moldy herbs is that the herbs are infected with mold, when the external temperature and humidity (20 ∼ 35℃, relative humidity > 75 %) is suitable, the mold is breeding and reproducing to produce mold. Molds are most likely to multiply when the moisture content of herbs is higher than 15 %. It is said that each year due to mold, insect infestation of thousands of millions for the production of 20 to 30 percent [117]. In order to maintain the original quality of Chinese herbs and ensure the therapeutic effect, it is necessary to apply scientific care during the storage process. Therefore the research on finding a safe, scientific, economical and appropriate methodology for the prevention of mold in Chinese herbal medicines can lay a quality foundation for the development, production and application of Chinese herbal medicines. The microbial inactivation of ultrasound is usually attributed to cavitation effects, such as strong localized pressure and temperature pulse conditions as well as high intensity shear and turbulence. These cavitation effects can lead to cell wall rupture and DNA damage, ultimately leading to the inactivation of different microorganisms [118].

It is reported that high-power intensity ultrasound can cause damage to microbial cell membranes that cannot be repaired, and cell membrane damage directly causes cytoplasmic loss and metabolic disorders. Cao et al. [119] used ultrasonic treatment to treat barley grass for 10 min. Then the soaked barley straw was freeze-dried under vacuum-0.09 MPa. Compared with the untreated condition, the ultrasonic wave with a frequency of 60 W/L reduced the total number of colonies by 33 %. This greatly improves the stability and storage period of barley grass.

In order to further improve the effect of ultrasonic sterilization, it is essential to select the appropriate technology combined with ultrasonic. Li [120] carried out ordinary drying, ultrasonic treatment and ultrasonic-assisted chitosan solution treatment on Astragalus membranaceus. The stable storage period of A.membranaceus after treatment was 5,12 and 24 months, respectively. The results showed that ultrasonic-assisted chitosan solution treatment could significantly increase the storage time of Astragalus membranaceus. The reason may be that the microorganisms and eggs on the surface were completely removed during the ultrasonic treatment. This eliminates the potential risks of mildew and insect infestation of Chinese medicinal materials in advance. Then, chitosan was used to isolate the medicinal materials from the external environment by ultrasonic dispersion.

Thermal ultrasonic drying technology refers to the combination of ultrasound and temperatures below 100 (usually 50℃ and 60℃). Studies have shown that thermal ultrasonic drying technology can reduce aerobic mesophilic bacteria in materials (reduced by 1.42–3.54 log CFU/mL), and even completely inactivate Enterobacteriaceae. It is worth noting that compared with the pasteurization method, the total phenol content of *Annona muricata* after hot ultrasonic drying was increased by 50 % [121]. In addition, the antioxidant activity (ABTS^·+^,DPPH^·^ and FRAP) was also improved to a certain extent. The reason for the increase in antioxidant activity may be that ultrasound will produce hydroxylation at the ortho or para position of the aromatic ring of phenolic compounds. In general, the combination of ultrasonic sterilization technology and other technologies can further sterilize Chinese herbal medicines. It can delay the mildew rate of Chinese herbal medicine and prolong the storage time. This has guiding significance for the practical application of ultrasonic technology in the anti-mildew and sterilization of Chinese medicinal materials.

Ultrasonic treatment of Chinese herbal medicines can eliminate hidden dangers in advance, which is a scientific and green Chinese herbal medicine maintenance technology. And ultrasonic and its combination technology can improve the green anti-mildew and mothproof effect of Chinese medicinal materials. At present, ultrasonic sterilization is widely used in the food industry, but there are few reports on the application of ultrasonic sterilization in the sterilization of Chinese herbal medicines. Therefore, ultrasonic sterilization of Chinese herbal medicine has broad research and application prospects, which is of great significance to promote the stable and rapid development of Chinese herbal medicine industry.

## 4 Conclusion

In the past 20 years, more and more literatures have shown that the application of ultrasound has been paid more and more attention by researchers and institutions around the world. At present, ultrasound is widely used in various processes of Chinese herbal medicine production and processing due to its physical and chemical effects, which helps to improve processing efficiency and reduce the time required for various processing operations. This technology has proved its ability in the extraction, cleaning, drying and sterilization of the active ingredients of Chinese medicinal materials. Whether ultrasound is used alone or in combination with other processing methods, it has a significant positive effect on the quality of Chinese medicinal materials. It not only reduces the damage caused by traditional processing technology to Chinese medicinal materials, but also improves the bioactive components and pharmacological activities of Chinese medicinal materials. Therefore, the application and research of ultrasound in the production and processing of traditional Chinese medicine are worthy of attention. With the continuous deepening of the research on the application field of ultrasound, improving the effect of processing and processing of Chinese herbal medicines, maintaining the original quality of Chinese herbal medicines, and improving the production efficiency of Chinese herbal medicines may be the current and future research hotspots. The application and promotion of ultrasonic technology is very important for the research and development of traditional Chinese medicine products, which meets the needs of the operation and sustainable development of traditional Chinese medicine industry. This will lay a quality foundation for the development, production and application of traditional Chinese medicine, thus accelerating the process of modernization and internationalization of traditional Chinese medicine.

## 5 Author agreement statement

We all the coauthors declare that this manuscript is original, has not been published before and is not currently being considered for publication elsewhere. We confirm that the manuscript has been read and approved by all named authors and that there are no other persons who satisfied the criteria for authorship but are not listed. We further confirm that the order of authors listed in the manuscript has been approved by all of us. We understand that the Corresponding Author is the sole contact for the Editorial process. He/she is responsible for communicating with the other authors about progress, submissions of revisions and final approval of proofs.

## CRediT authorship contribution statement

**Ke Yang:** Writing – review & editing, Writing – original draft, Resources, Funding acquisition. **Tao-hong Han:** Writing – review & editing, Writing – original draft, Software, Formal analysis, Data curation. **Yi-jun Liu:** Writing – review & editing, Writing – original draft, Software, Data curation. **Jia-ning Zhang:** Writing – review & editing, Writing – original draft, Software, Data curation. **Ping Zhou:** Writing – review & editing, Supervision, Resources, Funding acquisition, Data curation. **Xiao-ping Yu:** Writing – review & editing, Supervision, Resources, Funding acquisition, Data curation.

## Declaration of competing interest

The authors declare that they have no known competing financial interests or personal relationships that could have appeared to influence the work reported in this paper.

## References

[1] Sango D.M., Abela D., McElhatton A., Valdramidis V.P. (2014). Assisted ultrasound applications for the production of safe foods. J Appl Microbiol.

[2] Tan W.K., Cheah S.C., Parthasarathy S., Rajesh R.P., Pang C.H., Manickam S. (2021). Fish pond water treatment using ultrasonic cavitation and advanced oxidation processes. Chemosphere.

[3] Wang N.N., Li L.W., Wang K., Huang X.T., Han Y.H., Ma X.J., Wang M.H., Lv X., Bai X.M. (2023). Study and Application Status of Ultrasound in Organic Wastewater Treatment. Sustainability-Basel.

[4] Xiouras C., Fytopoulos A., Jordens J., Boudouvis A.G., Van Gerven T., Stefanidis G.D. (2018). Applications of ultrasound to chiral crystallization, resolution and deracemization. Ultrason Sonochem.

[5] Safari J., Banitaba S.H., Khalili S.D. (2012). Ultrasound-promoted an efficient method for one-pot synthesis of 2-amino-4,6-diphenylnicotinonitriles in water: a rapid procedure without catalyst. Ultrason Sonochem.

[6] Perez A., Kelley D.H. (2015). Ultrasound Velocity Measurement in a Liquid Metal Electrode. J vis Exp.

[7] C.M. Schoellhammer, A. Schroeder, R. Maa, G.Y. Lauwers, A. Swiston, M. Zervas, R. Barman, A.M. DiCiccio, W.R. Brugge, D.G. Anderson, D. Blankschtein, R. Langer, G. Traverso, Ultrasound-mediated gastrointestinal drug delivery, Sci Transl Med, 7 (2015) 310ra168. 10.1126/scitranslmed.aaa5937 PMC4825174 26491078

[8] Bi D., Shi L., Li B., Li Y., Liu C., Le L.H., Luo J., Wang S., Ta D. (2024). The Protocol of Ultrasonic Backscatter Measurements of Musculoskeletal Properties. Phenomics.

[9] Paniwnyk L. (2017). Applications of ultrasound in processing of liquid foods: A review. Ultrason Sonochem.

[10] Zheng K., Wang Y., Wang X., Zhu T., Chen X., Zhao Y., Sun P., Tong Y., Liu Y. (2023). Enhanced methane production from anaerobic digestion of waste activated sludge by combining ultrasound with potassium permanganate pretreatment. Sci Total Environ.

[11] Yan Y., Zhang Y., Wan J., Gao J., Liu F. (2023). Optimization of protein recovery from sewage sludge via controlled and energy-saving ultrasonic-alkali hydrolysis. Sci Total Environ.

[12] Carrillo-Lopez L.M., Garcia-Galicia I.A., Tirado-Gallegos J.M., Sanchez-Vega R., Huerta-Jimenez M., Ashokkumar M., Alarcon-Rojo A.D. (2021). Recent advances in the application of ultrasound in dairy products: Effect on functional, physical, chemical, microbiological and sensory properties. Ultrason Sonochem.

[13] Shriki J. (2014). Ultrasound Physics. Crit. Care Clin..

[14] Vardanega R., Santos D.T., Meireles M.A. (2014). Intensification of bioactive compounds extraction from medicinal plants using ultrasonic irradiation. Pharmacogn Rev.

[15] Cheng Y.X., Liu Z.Y., Xu B., Song P.P., Chao Z.M. (2023). Research status of insect infestation of Chinese medicinal materials during storage. Zhongguo Zhong Yao Za Zhi.

[16] Ying G.Y., Zhao X., Wang J.L., Yang M.H., Guo W.Y., Kong W.J. (2016). Application and prospect of “couplet medicine” techniques in preservation of Chinese medicinal materials. Zhongguo Zhong Yao Za Zhi.

[17] Chen L.W., K.M., Zhu Y.H., Cai H., Cai B. (2015). Research status and prospect of primary processing of traditional Chinese medicinal materials. China Journal of Chinese Materia Medica.

[18] Duan J., Su S., Lv J., Yan H., Ding A. (2009). Traditional experiences and modern cognition on primary processing of traditional Chinese medicinal materials. Zhongguo Zhong Yao Za Zhi.

[19] Zhu Y.H., Hu P., Luo Y.X., Yao X.Q. (2024). Knowledge mapping of trends and hotspots in the field of exercise and cognition research over the past decade. Aging Clin Exp Res.

[20] Shen L.P., Pang S.X., Zhong M.M., Sun Y.F., Qayum A., Liu Y.X., Rashid A., Xu B.G., Liang Q.F., Ma H.L., Ren X.F. (2023). A comprehensive review of ultrasonic assisted extraction (UAE) for bioactive components: Principles, advantages, equipment, and combined technologies. Ultrason. Sonochem..

[21] Shang H., Wang M., Li R., Duan M., Wu H., Zhou H. (2018). Extraction condition optimization and effects of drying methods on physicochemical properties and antioxidant activities of polysaccharides from Astragalus cicer L. Sci Rep.

[22] Yin H.M., Zhang Y.L., Hu T.T., Li W., Deng Y., Wang X., Tang H.Q., Zhao L., Yan G.W. (2023). Optimization of Cellulase-Assisted Extraction of Total Flavonoids from Equisetum via Response Surface Methodology Based on Antioxidant Activity. Processes.

[23] Li L., Yang X.Y., Pan L., Su Y., Wang Y. (2019). Comparing three Methods of Extraction of Auricularia Auricula Polysaccharides. Current Topics in Nutraceutical Research.

[24] Boitsova T.A., Brovko O.S., Ivakhnov A.D., Zhil'tsov D.V. (2020). Optimizing Supercritical Fluid Extraction of Usnic Acid from the Lichen Species. Russ J Phys Chem b+.

[25] Meng H., Wu J., Shen L., Chen G., Jin L., Yan M., Wan H., He Y. (2022). Microwave assisted extraction, characterization of a polysaccharide from Salvia miltiorrhiza Bunge and its antioxidant effects via ferroptosis-mediated activation of the Nrf2/HO-1 pathway. Int J Biol Macromol.

[26] Luo X., Gong H., He Z., Zhang P., He L. (2021). Recent advances in applications of power ultrasound for petroleum industry. Ultrason Sonochem.

[27] Ullah S., Khalil A.A., Shaukat F., Song Y.D. (2019). Sources, Extraction and Biomedical Properties of Polysaccharides. Foods.

[28] Leong Y.K., Yang F.C., Chang J.S. (2021). Extraction of polysaccharides from edible mushrooms: Emerging technologies and recent advances. Carbohydr Polym.

[29] Mtetwa M.D., Qian L., Zhu H., Cui F., Zan X., Sun W., Wu D., Yang Y. (2020). Ultrasound-assisted extraction and antioxidant activity of polysaccharides from Acanthus ilicifolius. J. Food Meas. Charact..

[30] Kang Q., Chen S., Li S., Wang B., Liu X., Hao L., Lu J. (2019). Comparison on characterization and antioxidant activity of polysaccharides from Ganoderma lucidum by ultrasound and conventional extraction. Int J Biol Macromol.

[31] Li W., Zhang Y., Zhao X., Fang L., Yang T., Xie J. (2023). Optimization of ultrasonic-assisted extraction of Platycodon grandiflorum polysaccharides and evaluation of its structural, antioxidant and hypoglycemic activity. Ultrason Sonochem.

[32] Huang D., Xia Q., Li F., Yang W., Nie S., Xie M. (2018). Attenuation of intestinal inflammation of polysaccharides from the seeds of Plantago asiaticaL. as affected by ultrasonication. J. Food Biochem..

[33] Wei Q., Zhang Y.H. (2023). Ultrasound-assisted polysaccharide extraction from Cercis chinensis and properites, antioxidant activity of polysaccharide. Ultrason Sonochem.

[34] Guo L., Kong N., Zhang X., Ma H. (2022). Multimode ultrasonic extraction of polysaccharides from maca (Lepidium meyenii): Optimization, purification, and in vitro immunoregulatory activity. Ultrason Sonochem.

[35] Yu W., Li J., Xiong Y., Wang J., Liu J., Baranenko D., Zhang Y., Lu W. (2024). Optimization of ultrasound-assisted extraction of Imperata cylindrica polysaccharides and evaluation of its anti-oxidant and amelioration of uric acid stimulated cell apoptosis. Ultrason Sonochem.

[36] Hu J., Jia X., Fang X., Li P., He C., Chen M. (2016). Ultrasonic extraction, antioxidant and anticancer activities of novel polysaccharides from Chuanxiong rhizome. Int J Biol Macromol.

[37] Liu M., Lu W., Ku K.M., Zhang L., Lei L., Zong W. (2020). Ultrasonic-assisted extraction and antioxidant activity of polysaccharides from Eucommia ulmoides leaf. Pak J Pharm Sci.

[38] Hu X., Xu F., Li J., Li J., Mo C., Zhao M., Wang L. (2022). Ultrasonic-assisted extraction of polysaccharides from coix seeds: Optimization, purification, and in vitro digestibility. Food Chem.

[39] Tian S., Hao C., Xu G., Yang J., Sun R. (2017). Optimization conditions for extracting polysaccharide from Angelica sinensis and its antioxidant activities. J Food Drug Anal.

[40] Meng Q., Chen Z., Chen F., Zhang Z., Gao W. (2021). Optimization of ultrasonic-assisted extraction of polysaccharides from Hemerocallis citrina and the antioxidant activity study. J Food Sci.

[41] Brglez Mojzer E., Knez Hrncic M., Skerget M., Knez Z., Bren U. (2016). Polyphenols: Extraction Methods, Antioxidative Action, Bioavailability and Anticarcinogenic Effects. Molecules.

[42] Oroian M., Ursachi F., Dranca F. (2020). Ultrasound-Assisted Extraction of Polyphenols From Crude Pollen. Antioxidants (basel).

[43] Yu Y., Wang L., Fu X., Wang L., Fu X., Yang M., Han Z., Mou H., Jeon Y.-J. (2019). Anti-oxidant and anti-inflammatory activities of ultrasonic-assistant extracted polyphenol-rich compounds from Sargassum muticum. Journal of Oceanology and Limnology.

[44] Garofulic I.E., Zoric Z., Pedisic S., Brncic M., Dragovic-Uzelac V. (2018). UPLC-MS Profiling of Blackthorn Flower Polyphenols Isolated by Ultrasound-Assisted Extraction. J. Food Sci..

[45] Kunjiappan S., Panneerselvam T., Govindaraj S., Kannan S., Parasuraman P., Arunachalam S., Sankaranarayanan M., Baskararaj S., Palanisamy P., Ammunje D.N. (2019). Optimization and analysis of ultrasound-assisted extraction of bioactive polyphenols from Garcinia indica using RSM and ANFIS modeling and its anticancer activity. J. Iran. Chem. Soc..

[46] Zhang S., Xie H., Huang J., Chen Q., Li X., Chen X., Liang J., Wang L. (2024). Ultrasound-assisted extraction of polyphenols from pine needles (Pinus elliottii): Comprehensive insights from RSM optimization, antioxidant activity. UHPLC-Q-Exactive Orbitrap MS/MS Analysis and Kinetic Model, Ultrason Sonochem.

[47] Sun Y., Lu J., Li J., Li P., Zhao M., Xia G. (2023). Optimization of ultrasonic-assisted extraction of polyphenol from Areca nut (Areca catechu L.) seeds using response surface methodology and its effects on osteogenic activity. Ultrason Sonochem.

[48] Gonzalez-Silva N., Nolasco-Gonzalez Y., Aguilar-Hernandez G., Sayago-Ayerdi S.G., Villagran Z., Acosta J.L., Montalvo-Gonzalez E., Anaya-Esparza L.M. (2022). Ultrasound-Assisted Extraction of Phenolic Compounds from Psidium cattleianum Leaves: Optimization Using the Response Surface Methodology. Molecules.

[49] Gueffai A., Gonzalez-Serrano D.J., Christodoulou M.C., Orellana-Palacios J.C., Ortega M.L.S., Ouldmoumna A., Kiari F.Z., Ioannou G.D., Kapnissi-Christodoulou C.P., Moreno A., Hadidi M. (2022). Phenolics from Defatted Black Cumin Seeds (Nigella sativa L.): Ultrasound-Assisted Extraction Optimization, Comparison, and Antioxidant Activity. Biomolecules.

[50] Irakli M., Chatzopoulou P., Ekateriniadou L. (2018). Optimization of ultrasound-assisted extraction of phenolic compounds: Oleuropein, phenolic acids, phenolic alcohols and flavonoids from olive leaves and evaluation of its antioxidant activities. Ind. Crop. Prod..

[51] Nafar M., Emam-Djomeh Z., Yousefi S., Ravan M.H. (2013). An Optimization Study on the Ultrasonic Treatments for Inactivation in Red Grape Juice with Maintaining Critical Quality Attributes. J Food Quality.

[52] Upadhyay R., Nachiappan G., Mishra H.N. (2015). Ultrasound-assisted extraction of flavonoids and phenolic compounds from leaves. Food Sci. Biotechnol..

[53] Hu D.B., Xue R., Zhuang X.C., Zhang X.S., Shi S.L. (2023). Ultrasound-assisted extraction optimization of polyphenols from Boletus bicolor and evaluation of its antioxidant activity. Front Nutr.

[54] Chen Y., Sun X., Fang L., Jiang X., Zhang X., Ge Z., Wang R., Wang C. (2022). Optimization of Ultrasound-Assisted Extraction of Polyphenols from Ilex latifolia Using Response Surface Methodology and Evaluation of Their Antioxidant Activity. Molecules.

[55] Dai K., Wei Y., Jiang S., Xu F., Wang H., Zhang X., Shao X. (2021). Lab Scale Extracted Conditions of Polyphenols from Thinned Peach Fruit Have Antioxidant, Hypoglycemic, and Hypolipidemic Properties. Foods.

[56] Luo Q., Zhang J.R., Li H.B., Wu D.T., Geng F., Corke H., Wei X.L., Gan R.Y. (2020). Green Extraction of Antioxidant Polyphenols from Green Tea (Camellia sinensis). Antioxidants (basel).

[57] Chmelova D., Skulcova D., Legerska B., Hornik M., Ondrejovic M. (2020). Ultrasonic-assisted extraction of polyphenols and antioxidants from Picea abies bark. J Biotechnol.

[58] Chávez-González M.L., Sepúlveda L., Verma D.K., Luna-García H.A., Rodríguez-Durán L.V., Ilina A., Aguilar C.N. (2020). Conventional and Emerging Extraction Processes of Flavonoids. Processes.

[59] Wen Y., Zeng X., Dai H., Liu B. (2023). Optimization of ultrasonic assisted extraction and biological activity of total flavonoids from Ligusticum chuanxiong Hort. using response surface methodology. Biomass Convers. Biorefin..

[60] Chaves J.O., de Souza M.C., da Silva L.C., Lachos-Perez D., Torres-Mayanga P.C., Machado A., Forster-Carneiro T., Vazquez-Espinosa M., Gonzalez-de-Peredo A.V., Barbero G.F., Rostagno M.A. (2020). Extraction of Flavonoids From Natural Sources Using Modern Techniques. Front Chem.

[61] Zuo L., Ao X., Guo Y. (2020). Study on the synthesis of dual-chain ionic liquids and their application in the extraction of flavonoids. J Chromatogr A.

[62] D. Tungmunnithum, S. Drouet, A. Kabra, C. Hano, Enrichment in Antioxidant Flavonoids of Stamen Extracts from Nymphaea lotus L. Using Ultrasonic-Assisted Extraction and Macroporous Resin Adsorption, Antioxidants (Basel), 9 (2020). 10.3390/antiox9070576 PMC7402147 32630721

[63] Vo T.P., Nguyen N.T.U., Le V.H., Phan T.H., Nguyen T.H.Y., Nguyen D.Q. (2023). Optimizing Ultrasonic-Assisted and Microwave-Assisted Extraction Processes to Recover Phenolics and Flavonoids from Passion Fruit Peels. ACS Omega.

[64] Zhao Z., Liu P., Wang S., Ma S. (2017). Optimization of ultrasound, microwave and Soxhlet extraction of flavonoids from Millettia speciosa Champ. and evaluation of antioxidant activities in vitro. J. Food Meas. Charact..

[65] Liang Q., Chen H., Zhou X., Deng Q., Hu E., Zhao C., Gong X. (2017). Optimized microwave-assistant extraction combined ultrasonic pretreatment of flavonoids from Periploca forrestii Schltr. and evaluation of its anti-allergic activity. Electrophoresis.

[66] Vo T.P., Tran T.Q.D., Phan T.H., Huynh H.D., Vo T.T.Y., Vo N.M.K., Ha M.P., Le T.N., Nguyen D.Q. (2023). Ultrasonic-assisted and enzymatic-assisted extraction to recover tannins, flavonoids, and terpenoids from used tea leaves using natural deep eutectic solvents. Int J Food Sci Tech.

[67] Yang R.F., Geng L.L., Lu H.Q., Fan X.D. (2017). Ultrasound-synergized electrostatic field extraction of total flavonoids from Hemerocallis citrina baroni. Ultrason Sonochem.

[68] Wu E.Y., Sun W.J., Wang Y., Zhang G.Y., Xu B.C., Chen X.G., Hao K.Y., He L.Z., Si H.B. (2022). Optimization of Ultrasonic-Assisted Extraction of Total Flavonoids from Abrus Cantoniensis (Abriherba) by Response Surface Methodology and Evaluation of Its Anti-Inflammatory Effect. Molecules.

[69] Liu C., Qin K., Qi Y., Li K., Li Y., Jia B. (2014). Optimization of ultrasonic extraction of total flavonoids from Tussilago farfara L. using response surface methodology. Pharmazie.

[70] Zhang H., Wang X., He D., Zou D., Zhao R., Wang H., Li S., Xu Y., Abudureheman B. (2021). Optimization of Flavonoid Extraction from Xanthoceras sorbifolia Bunge Flowers, and the Antioxidant and Antibacterial Capacity of the Extract. Molecules.

[71] Cui L., Ma Z., Wang D., Niu Y. (2022). Ultrasound-assisted extraction, optimization, isolation, and antioxidant activity analysis of flavonoids from Astragalus membranaceus stems and leaves. Ultrason Sonochem.

[72] Zeng X., Tan H., Liu B., Wen Y. (2023). Optimization of ultrasonic-assisted extraction and purification of total flavonoids with biological activities from Radix Puerariae. Biomass Convers. Biorefin..

[73] Liu Y., Luo X.L., Lan Z.Q., Tang J.R., Zhao P., Kan H. (2018). Ultrasonic-assisted extraction and antioxidant capacities of flavonoids from Camellia fascicularis leaves, Cyta-J. Food.

[74] Li F.R., Liu S.M., Liu F.X., Zhang S.Z., Sun Y.L., Ding J.M. (2019). Ultrasonic cleaning technology and its application in the cleaning of Chinese herbal medicines, Journal of Traditional Chinese. Vet. Med..

[75] Bilek S.E., Turantas F. (2013). Decontamination efficiency of high power ultrasound in the fruit and vegetable industry, a review. Int J Food Microbiol.

[76] Zhou W., Sarpong F., Zhou C. (2022). Use of Ultrasonic Cleaning Technology in the Whole Process of Fruit and Vegetable Processing. Foods.

[77] Bhargava N., Mor R.S., Kumar K., Sharanagat V.S. (2021). Advances in application of ultrasound in food processing: A review. Ultrason Sonochem.

[78] Yu Y., Wang Y., Okonkwo C.E., Chen L., Zhou C. (2024). Multimode ultrasonic-assisted decontamination of fruits and vegetables: A review. Food Chem.

[79] Rodriguez-Alonso J., Cabrales-Garcia C., Millan R. (2023). Factors influencing the cleaning of plant samples with ultrasonic technology. Int J Phytoremediation.

[80] Sun Z., Xu F., Tan H.J., Zhang J.Y. (2018). Study on Control Technology of Ultrasonic Herbs Cleaning Equipment, Value. Engineering.

[81] Chen J., Chen G.B., Liu S.M., Liu F.X., Li F.R. (2017). Washing Technology Research of Roots of Chinese Herbal Medicine Based on Ultrasonic. Journal of Minzu University of China.

[82] Huang Z.Y., Luo Y. (2024). Study on the analysis and detection method for the cleaning effect of ultrasonic cleaning of root Chinese herb Angelica sinensis. Laboratory Testing.

[83] Li F.R., Liu S.M., Liu F.X., Chen J. (2016). Preliminary experiment on ultrasonic cleaning of Chinese herbal medicine Angelica sinensis, Journal of Traditional Chinese. Vet. Med..

[84] Zhang L.J., Zha L., Fan B., Wang F.Z. (2018). Multi-factor test and parameters optimization of ultrasonic cleaning of gymnadenia conopsea r. br. Journal of Inner Mongolia Agricultural University.

[85] Alenyorege E.A., Ma H.L., Ayim I., Aheto J.H., Hong C., Zhou C.S. (2019). Effect of multi-frequency multi-mode ultrasound washing treatments on physicochemical, antioxidant potential and microbial quality of tomato. J. Food Meas. Charact..

[86] Qu Y.L., Guo L.N., Hong C., Wan Y.M., Tuly J., Ma H.L. (2023). Effects of multi-frequency ultrasonic assisted sodium hypochlorite on the cleaning effect and quality of fresh-cut scallion stems. Ultrason. Sonochem..

[87] Saifullah M., McCullum R., McCluskey A., Vuong Q. (2019). Effects of different drying methods on extractable phenolic compounds and antioxidant properties from lemon myrtle dried leaves. Heliyon.

[88] Thamkaew G., Sjoholm I., Galindo F.G. (2021). A review of drying methods for improving the quality of dried herbs. Crit Rev Food Sci Nutr.

[89] Fan K., Zhang M., Mujumdar A.S. (2017). Application of airborne ultrasound in the convective drying of fruits and vegetables: A review. Ultrason Sonochem.

[90] Huang D., Men K., Li D., Wen T., Gong Z., Sunden B., Wu Z. (2020). Application of ultrasound technology in the drying of food products. Ultrason Sonochem.

[91] Liu Y., Miao S., Sun Y., Li X., Zhu W. (2016). Drying Characteristics of Apple Slices during Contact Ultrasound Reinforced Heat Pump Drying, Transactions of the Chinese Society for Agricultural. Machinery.

[92] Jahanbakhshi A., Yeganeh R., Momeny M. (2020). Influence of ultrasound pre‐treatment and temperature on the quality and thermodynamic properties in the drying process of nectarine slices in a hot air dryer. J Food Process Pres.

[93] Oladejo A.O., Nkem O.M., Alonge A.F., Akpan M.G., Etti C.J., Okoko J.U., Etuk N.-N. (2023). Influence of ultrasound-pretreated convective drying of Roselle (Hibiscus sabdariffa L) leaves on its drying kinetics and nutritional quality. Sci. Afr..

[94] Zheng Q.R., Gao P.P., Liu T.T., Gao X.X., Li W.F., Zhao G.H. (2022). Effects of drying methods on colour, amino acids, phenolic profile, microstructure and volatile aroma components of Boletus aereus slices. Int J Food Sci Tech.

[95] Xu X.M., Wang N., Wang S.M., Wang J.Z., Wu N.N., Xu Y.D., Xu M. (2024). Effects of Different Pretreatments on Hot Air Drying Characteristics, Nutrition, and Antioxidant Capacity of Tartary Buckwheat Sprouts. Foods.

[96] Huang W.Y., Huang D., Qin Y.T., Zhang Y.X., Lu Y.J., Huang S., Gong G.L. (2023). Ultrasound-assisted hot air drying characteristics of Phyllanthus emblica. J. Food Process Eng.

[97] Sledz M., Wiktor A., Nowacka M., Witrowa-Rajchert D. (2015). Drying Kinetics, Microstructure and Antioxidant Properties of Basil Treated by Ultrasound. J. Food Process Eng.

[98] Pei Y., Li Z., Xu W., Song C., Li J., Song F. (2021). Effects of ultrasound pretreatment followed by far-infrared drying on physicochemical properties, antioxidant activity and aroma compounds of saffron (Crocus sativus L.). Food Biosci..

[99] Zhang D., Huang D., Zhang Y., Lu Y., Huang S., Gong G., Li L. (2023). Ultrasonic assisted far infrared drying characteristics and energy consumption of ginger slices. Ultrason Sonochem.

[100] Shang J., Zhang Q., Wang T., Xu Y., Zang Z., Wan F., Yue Y., Huang X. (2023). Effect of Ultrasonic Pretreatment on the Far-Infrared Drying Process and Quality Characteristics of Licorice. Foods.

[101] Ni J.B., Ding C.J., Zhang Y.M., Song Z.Q., Xu W.Q. (2020). Influence of ultrasonic pretreatment on electrohydrodynamic drying process of goji berry. J Food Process Pres.

[102] Yue Y., Zang Z., Wan F., Zhang Q., Shang J., Xu Y., Jiang C., Wang T., Huang X. (2022). Effect of Ultrasonic Pretreatment on Radio Frequency Vacuum Drying Characteristics and Quality of Codonopsis pilosula Slices. Agriculture.

[103] Jiang C., Wan F., Zang Z., Zhang Q., Ma G., Huang X. (2022). Effect of an Ultrasound Pre-Treatment on the Characteristics and Quality of Far-Infrared Vacuum Drying with Cistanche Slices. Foods.

[104] S.Y. Chao, L.D. Zhang, G.Z. Wang, L.K. Chen, J. Lu, Effect of ultrasonic pretreatment on microstructure and quality of hot-air dried begonia fruit (Calophyllum inophyllum L.) Food and Fermentation Industries Editorial Staff, 1-14.

[105] Fernandes F.A.N., Rodrigues S. (2023). Ultrasound applications in drying of fruits from a sustainable development goals perspective. Ultrason Sonochem.

[106] Tao Y., Li D., Siong Chai W., Show P.L., Yang X., Manickam S., Xie G., Han Y. (2021). Comparison between airborne ultrasound and contact ultrasound to intensify air drying of blackberry: Heat and mass transfer simulation, energy consumption and quality evaluation. Ultrasonics Sonochemistry.

[107] Wu J.X., Zhang L., Fan K. (2022). Recent advances in ultrasound-coupled drying for improving the quality of fruits and vegetables: a review. Int J Food Sci Tech.

[108] Zang Z., Zhang Q., Huang X., Jiang C., He C., Wan F. (2023). Effect of Ultrasonic Combined with Vacuum Far-infrared on the Drying Characteristics and Physicochemical Quality of Angelica sinensis. Food Bioproc. Tech..

[109] Wang T.X., Ying X.Y., Zhang Q., Xu Y.R., Jiang C.H., Shang J.W., Zang Z.P., Wan F.X., Huang X.P. (2024). Drying kinetics prediction and quality effect of ultrasonic synergy vacuum far-infrared drying of. J. Food Sci..

[110] Feng Z., Zhang M., Guo L., Shao R., Wang X., Liu F. (2024). Effect of Direct-Contact Ultrasonic and Far Infrared Combined Drying on the Drying Characteristics and Quality of Ginger. Processes.

[111] Vallespir F., Crescenzo L., Rodríguez O., Marra F., Simal S. (2019). Intensification of Low-Temperature Drying of Mushroom by Means of Power Ultrasound: Effects on Drying Kinetics and Quality Parameters. Food Bioproc. Tech..

[112] Wu B., Ma G., Wan F., Ma J., Zang Z., Xu Y., Chen A., Huang X. (2024). Effect of Ultrasound-Assisted Vacuum Far-Infrared on the Drying Characteristics and Qualities Attributes of Cistanche Slices. Agriculture.

[113] Nguyen H., Le Q.-H., Le T.-D., Pham V.-K. (2022). Experimental Research to Determine the Effect of Ultrasound in Drying Bo Chinh Ginseng by Ultrasound-Assisted Heat Pump Drying Method. Appl. Sci..

[114] Wang X., Xu S., Wu Z., Li Y., Wang Y., Wu Z., Zhu G., Yang M. (2022). A novel ultrasound-assisted vacuum drying technique for improving drying efficiency and physicochemical properties of Schisandra chinensis extract powder. Food Sci Nutr.

[115] Zhang Q., Wan F., Zang Z., Jiang C., Xu Y., Huang X. (2022). Effect of ultrasonic far-infrared synergistic drying on the characteristics and qualities of wolfberry (Lycium barbarum L.). Ultrason Sonochem.

[116] Liu Y., Sun Y., Miao S., Li F., Luo D. (2015). Drying characteristics of ultrasound assisted hot air drying of Flos Lonicerae. J Food Sci Technol.

[117] Li F.R., Liu S.M., Liu F.X., Han L.M., Zhang S.Z., Sun Y.L., Ding J.M. (2017). Preliminary Analysis Greening Mildew Proofing and Mothproofing of Traditional Chinese Medicinal Materials Based on Ultrasonic Hyphenated Techniques, Journal of Animal Science. Vet. Med..

[118] Khandpur P., Gogate P.R. (2016). Evaluation of ultrasound based sterilization approaches in terms of shelf life and quality parameters of fruit and vegetable juices. Ultrason Sonochem.

[119] Cao X., Zhang M., Mujumdar A.S., Zhong Q., Wang Z. (2018). Effects of ultrasonic pretreatments on quality, energy consumption and sterilization of barley grass in freeze drying. Ultrason Sonochem.

[120] Li F.R. (2018). Ultrasound-assisted Cleaning and Green Conservation of Astragalus Mongholicus. Journal of Minzu University of China.

[121] Zafra-Rojas Q.Y., Jimenez-Hernandez J.L., Olloqui E.J., Del Socorro Cruz-Cansino N., Alanis-Garcia E., Ramirez-Moreno E., Ariza-Ortega J.A., Moreno-Secena J.C. (2023). Optimization of Thermoultrasound Process of Soursop (Annona muricata) Nectar and Comparison of Its Physicochemical Properties and In Vitro Bioaccessibility of Antioxidants with Pasteurized Sample, Food Technol. Biotechnol.

